# Studies on decolorization of reactive blue 19 textile dye by *Coprinus plicatilis*

**DOI:** 10.1186/2052-336X-12-49

**Published:** 2014-02-24

**Authors:** Hatice A Akdogan, Merve C Topuz, Asiye A Urhan

**Affiliations:** 1 Department of Chemistry, Faculty of Science and Arts, Pamukkale University, Denizli, Turkey

**Keywords:** Reactive blue 19, White rot fungi, Decolorization, Metabolite, UV-vis

## Abstract

**Background:**

Studies were carried on the decolorization of the textile dye reactive blue 19 (RB 19) by a novel isolate of *Coprinus plicatilis* (*C. plicatilis*) fungi. We describe an in vitro optimization process for decolorization and its behavior under different conditions of carbon and nitrogen sources, pH, temperature and substrate concentration.

**Results:**

The optimal conditions for decolorization were obtained in media containing intermediate concentrations of ammonium oxalate and glucose (10 g/L) as nitrogen and carbon sources, respectively, at 26°C and pH = 5.5. Maximum decolorization efficiency against RB 19 achieved in this study was around 99%. Ultra-violet and visible (UV-vis) spectrophotometric analyses, before and after decolorization, suggest that decolorization was due to biodegradation.

**Conclusions:**

This effect was associated with laccase enzyme displaying good tolerance to a wide range of pH values, salt concentrations and temperatures, suggesting a potential role for this organism in the remediation of real dye containing effluents. In conclusion, laccase activity in *C. plicatilis* was firstly described in this study.

## Introduction

Treatment of synthetic dyes in wastewater is a matter of great concern. Several physical and chemical methods have been employed for the removal of dyes [1]. However, these procedures have not been widely used due to high cost, formation of hazardous by products and intensive energy requirement [2]. Worldwide over 10,000 different dyes and pigments are used in dyeing and printing industries. The total world colorant production is estimated to be 8.00.000 tons per year and at least 10% of the used dyestuff enters the environment through wastes [3,4]. Wastewater from textile industries constitutes a threat to the environment in many parts of the world. Although some of the dyes are not themselves toxic, after release into the aquatic environment their degradation products are often carcinogenic [5,6]. The existing technologies for decolorization of textile dyeing effluents like adsorption, precipitation, membrane filtration, chemical degradation and photochemical degradation are relatively expensive and commercially unattractive [7,8]. Coagulation-flocculation has major operational problems as it generates large amounts of sludge. Adsorption technique is also more expensive as it involves the use of powdered activated carbon as adsorbent and disposal of spent adsorbent [9] is still a problem. Microbial decolorization is a potential and an effective alternative for the decolorization of wastewater. Microbial decolorization methods of textile dye containing effluents have been reviewed and reported. White-rot fungi such as *Phanerochaete chrysosporium*[10,11], *Trametes versicolor*[12,13], *Bjerkandera adusta*[14], *Pycnoporus cinnabarinus*[15] and *Phanerochaete sordida*[16] have been shown to decolorize textile dyes or coloured effluents [17].

In recent years, the utilization of biodegradative abilities of some white rot fungi seems to be promising. They do not require preconditioning to particular pollutants and owing to their extracellular non-specific free radical-based enzymatic system they can degrade to nondetectable levels or even completely eliminate a variety of xenobiotics including synthetic dyes. Many white rot fungi (*Phanerochaete chrysosporium*, *Pleurotus ostreatus*, *Bjerkandera adusta*, *Trametes versicolor*, etc.) have been intensively studied in connection with their ligninolytic enzyme production and their decolorization ability [18-25]. This capability is due to extracellular non-specific and non-stereoselective enzyme systems composed of laccases (EC 1.10.3.2), lignin peroxidases (EC 1.11.10.14) and manganese peroxidases (EC 1.11.1.13) [26]. Laccase based decolorization treatments are potentially advantageous to bioremediation Technologies since the enzyme is produced in larger amounts mainly by numerous fungi [27,28]. Laccase belongs to a family of multi-copper oxidases that are widespread in nature. Laccases are related to oxidation of a range of aromatic, toxic and environmentally problematic substrates [29] and particular interest with industrial applications. The potential applications are in the textile industry [30], detoxification of pollutants and industrial effluents [31], pulp and paper industry [32], food and pharmaceutical industries [33] biosensor and biofuel applications [34].

The aim of the present work was to characterize the biodegradation of the textile dye Remazol reactive blue 19, by the action of soluble extracts from the white rot fungus *C. plicatilis*. We describe an in vitro optimization process for decolorization and its behavior under different conditions of carbon and nitrogen sources, pH, temperature and substrate concentration.

## Materials and methods

### Dyes and chemicals

Textile Remazol dye: reactive blue 19 (RB 19) was supplied by Dystar (Kocaeli, Turkey). 2,2-Azino-bis (3-ethylbenzothiazoline-6-sulfonic acid) (ABTS) was obtained from Sigma Chemical Company (St. Louis, MO, USA). All chemicals used were of the highest purity available and of analytical grade.

### Culture conditions

Mycelial suspension of *Coprinus plicatilis* isolated in our university fungus research Laboratory. The white rot fungi *Coprinus plicatilis (C. plicatilis)* were maintained on 2% (w/v) malt agar slants at 4°C and were then activated at 26°C for 3 days. The mycelium were harvested with a sterile 0.9% NaCl solution and were then inoculated into 100 mL of 2% malt extract broth (pH = 4.5) in 250 mL Erlenmeyer flasks at 26°C and 175 rpm for 4 days. Pellets were inoculated into the medium consisting of 10 g/L glucose, 1.0 g/L of NH_4_H_2_PO_4_, 0.05 g/L of MgSO_4_.7H_2_O, 0.01 g/L of CaCl_2_, 0.025 g/L of yeast extract. Cultivation was carried out in an orbital shaker incubator, at 26°C, 175 rpm [35]. At the beginning of the fourth day of incubation, dye solution was added to the flasks, aseptically, at desired concentrations. Aliquots were assayed for laccase activity. Experiments were performed in 250 mL Erlenmeyer flasks containing 50 mL of liquid medium. For biomass calculation, mycelia were filtered through previously dried and tared Whatman No. 1 filter papers, washed with distilled water and dried at 50°C to constant weight.

### Spectrophotometric analysis

Aliquots of 1–2 mL volume of clear dye solution were taken from each reaction flask at regular time intervals and measured immediately using a UV-vis recording double beam spectrophotometer (Shimadzu UV-1601). Decolorization was determined spectrophotometrically by monitoring the absorbance at the wavelength maximum for this dye, and by the reduction of the major peak area in the visible region for dye. The percentage of in culture decolorization was calculated as;

Decolorization percentage, R (%)

(1) $$R=\left[\frac{\left(C_{o}-C\right)}{C_{o}}\right].100\;$$

Biodegradation Rate = (*C*_0_-*C*)/Incubation day

*C*_0_: Initial concentration of dye, *C*: Last concentration of dye

### Optimization of carbon and nitrogen content for enzyme production

Six different carbon substrates (glucose, sucrose, starch, maltose, fructose and glycerol) and nitrogen sources (sodium nitrate, urea, ammonium tartrate, ammonium carbonate, ammonium oxalate and peptone) were used to substitute the original carbon and nitrogen sources. The assessed concentrations were 5, 10 and 15 g/L. All inoculated media were incubated for 15 days at 26°C in the dark and the supernatant or eluted extracts collected for further analysis.

### Effect of pH, temperature and copper supplementation on enzyme production

Cultures optimized for carbon and nitrogen content were supplemented with copper sulfate (0.1-5 mM) and incubated for 15 days at 26°C. Supernatants or soluble extracts were then submitted to decolorization. Additionally, destaining activity was measured in samples derived from cultures grown at 26, 30 and 35°C for 15 days and at pH values from 5.5 to 7.0 at 26°C for 15 days.

### Enzyme assays

Laccase (Lac) (EC 1.10.3.2) production was assessed by a measurement of the enzymatic oxidation of 2, 2^2^-azinobis-(3 ethylbenzothiazoline- 6-sulphonic acid) (ABTS) at 420 nm (ϵ = 3.6 × 10^4^ cm^-1^ M^-1^) [36]. The reaction mixture contained 300 μL of extracellular fluid, 300 μL of 1 μM ABTS and 0.1 M Na Acetate buffer (pH = 4.5, 400 μL). One unit of enzyme activity is defined as the amount of enzyme that oxidizes 1 μmol ABTS in one minute.

### Effect of pH, temperature and salt concentration on decolorization

Soluble extracts derived from 15-day-old solid or liquid cultures, carried out under optimized conditions, were tested for decolorization. Decolorization of RB 19 (50 mg/L) was carried out at pH values from 2 to 9 (intervals of 0.5 units), adjusted by using 50 mM citrate-phosphate buffer. The temperatures assessed were 20, 30, 40, 50, 60 and 70°C. For testing susceptibility to salt, different NaCl concentrations (0.05, 0.1, 0.2, 0.4 and 0.6 M) were used in the assay.

### Statistical analysis

Standard deviations of the results of triplicate samples from the flask studies were calculated using the Microsoft Excel Spreadsheet Program.

## Results

### Screening using reactive dyes

**Figure 1:**
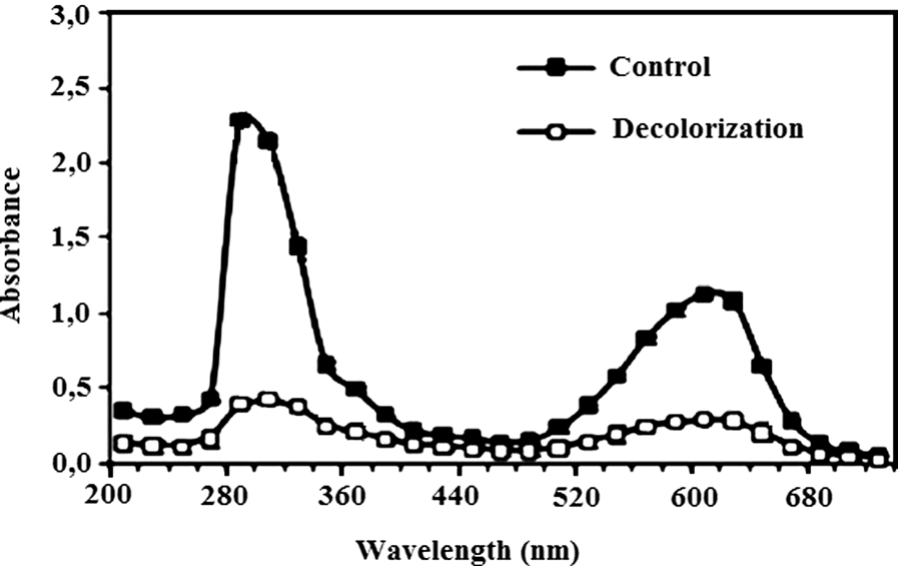
**UV-vis spectrum of a solution of RB19 before and after incubation with *C. plicatilis *** **cultures.**

*C. plicatilis* cultures were initially exposed to RB 19 textile dye. In culture decolorization occurred only with RB 19. The total flattening of the UV-vis spectra of the samples indicates that decolorization was accompanied by biodegradation since the smoothening of absorbance peaks at 600 and at 280 nm is consistent with the reduction of azo linkages and the loss of aromatic rings (Figure 1).

### Effect of dye concentration and water content on fungal growth

**Figure 2:**
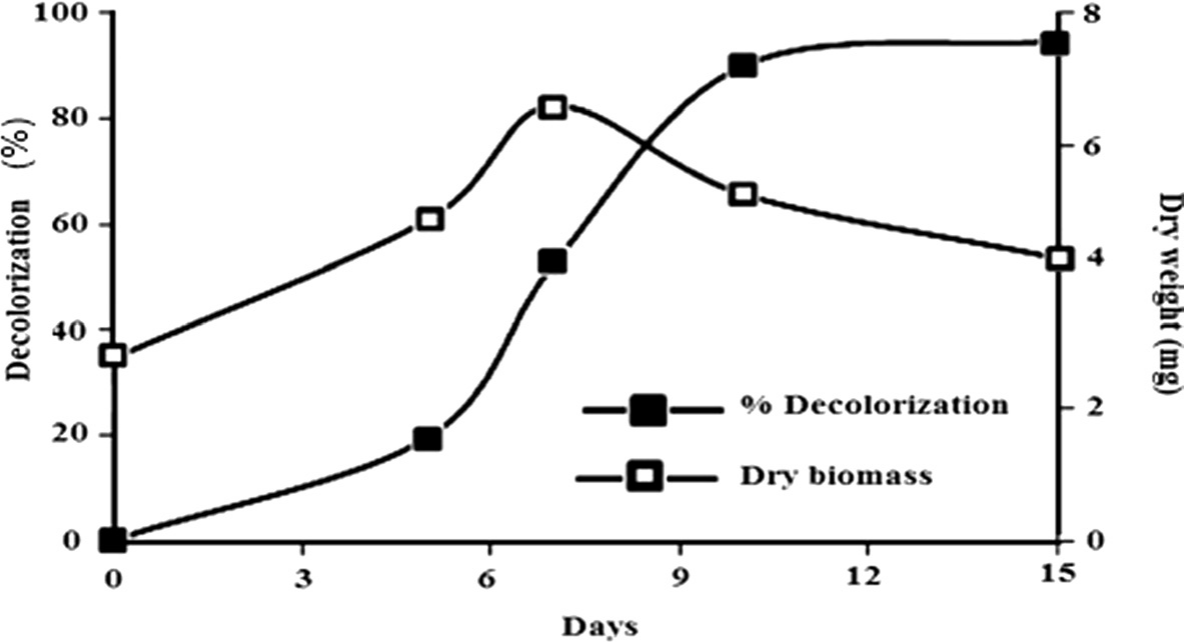
**Decolorization of RB19 and growth of *C. plicatilis* in liquid minimal medium containing 50 mg/L dye.** The absorbance of the culture supernatant was determined at 585 nm.

**Figure 3:**
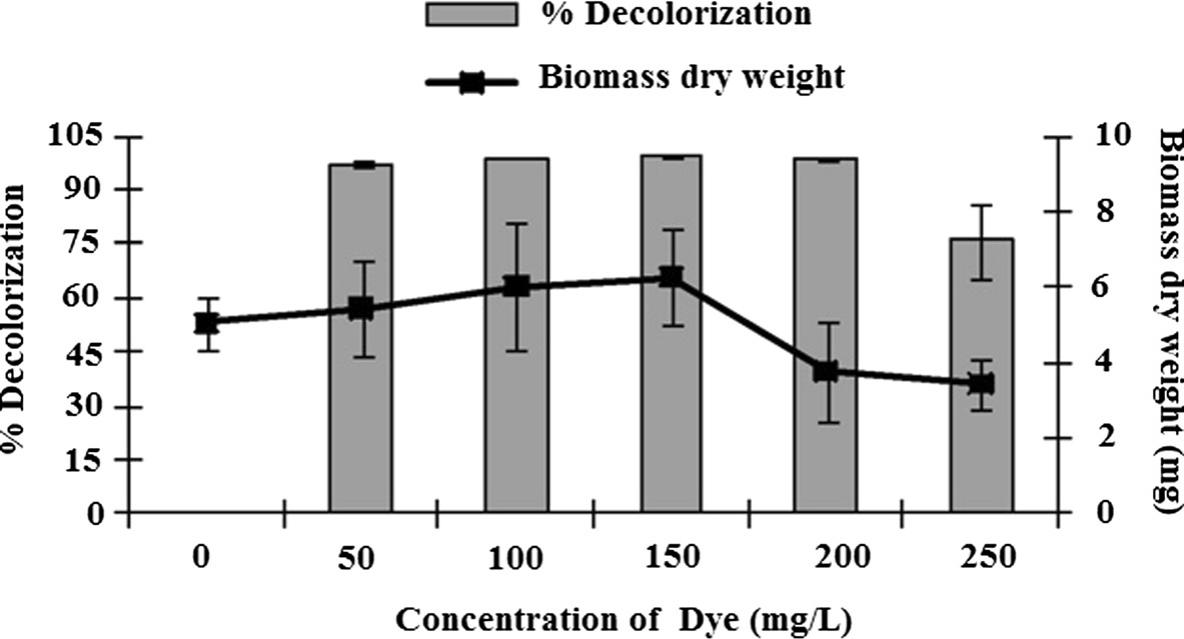
**Effect of dye concentration on fungal growth and in culture decolorization.** Absorbance at 585 nm was assessed after 15 days of culture.

When *C. plicatilis* was cultured in malt extract medium containing 50 mg/L RB 19, the percentage decolorization increased over time, reaching a peak of 99% decolorization after 15 days of incubation (Figure 2). The decolorization was not directly associated with fungal growth since over the period of 5–10 days the biomass level fell while the percentage decolorization was still increasing rapidly. In culture decolorization tests were then done with increasing concentrations of RB 19. Although these concentrations delayed fungal growth slightly, L. crinitus was able to develop in RB 19 concentrations as high as 200 or even 250 mg/L, giving percentage in culture decolorizations of 93.7% and 77.4%, respectively, after 15 days (Figure 3).

In order to determine whether the fungal products responsible for dye decolorization were secreted or mycelium-associated, supernatants and mycelia from dead liquid cultures and eluted extracts from dead solid cultures were subjected to decolorization. Decolorization was highest in supernatants and eluted extracts, with relatively low decolorization being detected in mycelia (results not shown). Decolorization eluted from solid cultures was very similar to that obtained in the supernatants obtained from liquid cultures. Also, an RB 19 concentration of 50 mg/L was selected. At this concentration the dye was totally consumed and slightly higher decolorizations were obtained in the culture extracts.

### Effect of carbon and nitrogen content on decolorization

**Figure 4:**
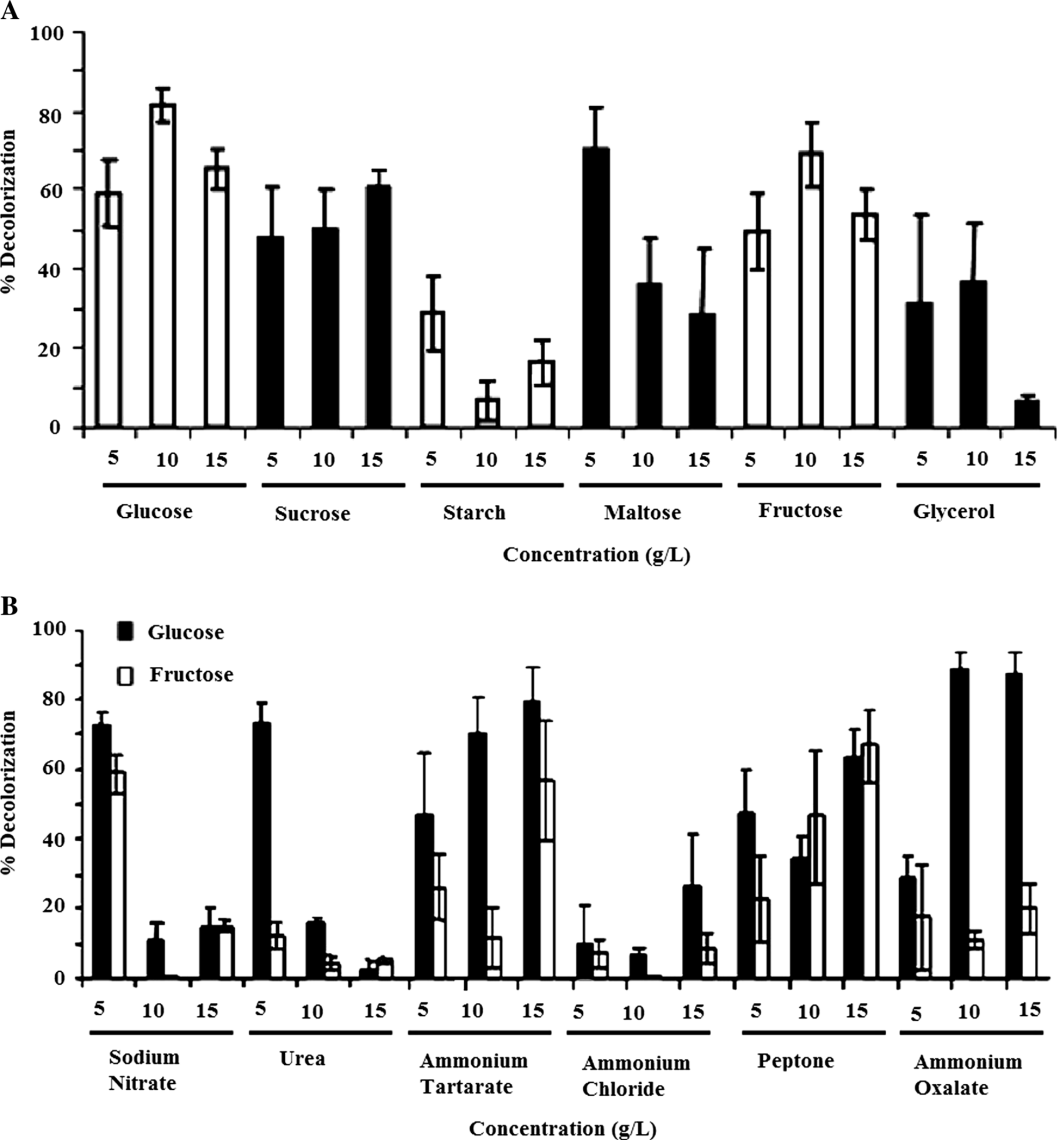
**Effect of carbon and nitrogen on decolorization. A**; Effect of carbon type and concentration on decolorization in solid medium. **B**; Effect of nitrogen source and concentraation on decolorization in solid media containing fructose (10 g/L) and glucose (10 g/L) as carbon source.

In the case of white rot fungi, it has been reported that the production of lignin modifying enzymes (LMEs) and decolorization varies greatly, according to the type and concentration of carbon and nitrogen sources in culture media. These effects were therefore investigated. *C. plicatilis* was grown on solid minimal media that contained glucose, fructose, maltose, starch, sucrose or glycerol as the sole carbon source. For each substrate, three concentrations (5, 10 and 15 g/L) were tested. After 15 days of incubation, decolorization was performed using extracts from the solid medium. Drastic differences in the decolorization were observed. *C. plicatilis* produced the highest levels of decolorization when grown in 10 g/L glucose; 5 g/L maltose or 10 g/L fructose, with the decolorization ranging from 63.7% to 84.8%. Higher concentrations of these carbon sources did not lead to higher decolorization. With some carbon sources the yield of decolorization was very low. For example, with 15 g/L glycerol and 10 g/L starch decolorization of only 5-15% were produced (Figure 4A).

When nitrogen sources were assessed, using fructose (10 g/L) and glucose (10 g/L) as carbon sources (maltose was omitted due to its high cost), soluble extracts derived from cultures containing 5 g/L sodium nitrate, 15 g/L ammonium tartrate and 10 g/L ammonium oxalate performed the highest decolorization (Figure 4B). Again, some substrates promoted the decolorization and some of them, such as urea and ammonium chloride, were inhibitory. A significant observation is that the effect of a nitrogen source can depend on the accompanying carbon substrate. Thus, soluble extracts derived from cultures containing 5 g/L urea led to a 10% decolorization of this dye when combined with 5 g/L fructose in the culture medium, but when combined with 5 g/L glucose, this same concentration of urea gave a 5-fold higher decolorization. A similar effect was observed for 10 and 15 g/L ammonium oxalate with fructose and glucose.

### Effect of pH, temperature and copper amount of cultures on decolorization of dye

Cultures were done in solid medium with optimized carbon and nitrogen contents (10 g/L glucose and 10 g/L ammonium oxalate) in five different initial pH values (at 26°C) and three different temperatures (with initial pH = 5.5). Other cultures were performed (at 26°C and initial pH = 5.5) in the presence of three different copper sulfate concentrations. Extracts from 4 day cultures were tested for decolorization. Decolorization of dye was better in extracts derived from cultures incubated at 26°C and with initial pH values ranging from 5.5 to 6.5 (Table 1). Extracts derived from copper supplemented cultures did not display an improvement in decolorization and at copper sulfate concentrations above 1 mM the decolorization was relatively low (Table 1).

**Table 1:** Effect of pH, temperature and copper concentration on decolorization (initial dye concentration; 50 mg/L)

**T°C % Decolorization**	**26** 95.1 ± 0.6	**30** 21.4 ± 2.3	**35** 0.04 ± 0.04	
**Cu**^**2+ **^**(mM) % Decolorization**	**0** 79.7 ± 2.2	**0.1** 84.3 ± 3.6	**1.0** 51.2 ± 1.3	**2.0** 49.6 ± 1.1
**pH % Decolorization**	**5.5** 99.2 ± 0.6	**6** 81.3 ± 1.2	**6.5** 68.7 ± 2.4	**7** 11.4 ± 1.7

### Laccase activities

**Figure 5:**
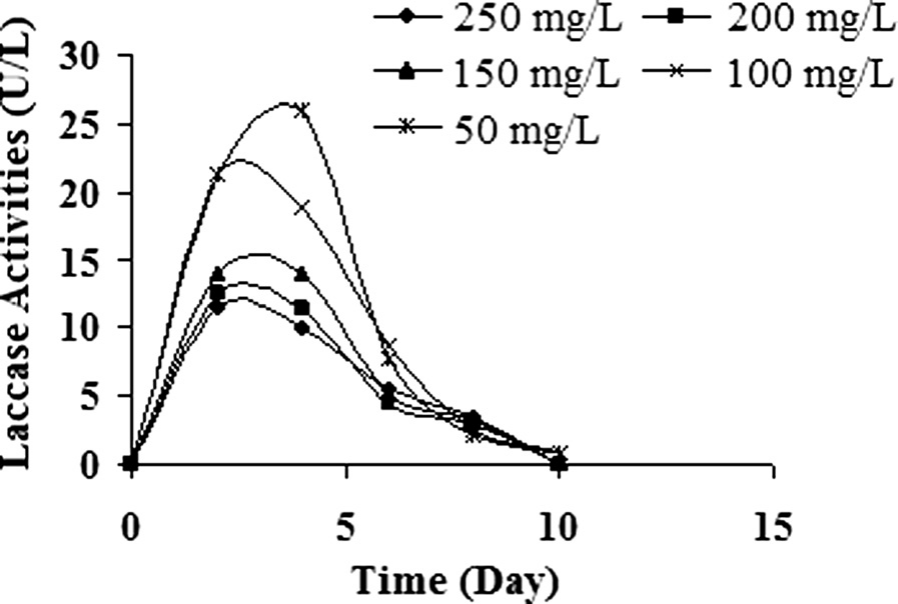
Alteration of laccase enzyme activities.

Figure 5 shows variations of laccase activities during decolorization within the ten days period for this organism. In an attempt to determine the possible role of ligninolytic enzymes on dye decolorization, laccase enzyme activity was monitored during the decolorization of this dye. In dye containing liquid cultures, laccase specific activity of this fungi was observed to have decreased by increasing dye concentration. These results emphasized the role of laccase in RB 19 decolorization. At higher concentrations levels than 50 mg/L RB 19, considered enzyme activities were diminished sharply, which is likely due to the toxicity of synthetic dyes as mentioned before (Figure 5).

## Discussions

Several species and strains have been assessed for biodegradation of different pollutants such as crude oil [37], pentachlorophenol [38], DDT [39], trinitrotoluene [40] and some textile dyes [41,42]. Here we report laccase activity in *C. plicatilis*, a relatively unexplored *Coprinus* species, and its participation in the decolorization of the textile dye reactive blue 19. Reactive blue 19 (RB 19) is a vinyl sulfone azo dye. Laccase activity in *C. plicatilis* was firstly described. Copper supplementation can stimulate laccase synthesis since the enzyme uses copper as cofactor, but the ion can also inhibit the growth of the organism [43]. Previous attempts to degrade it have focused on photo-catalytic and chemo-oxidative processes [44-46]. Although these processes may degrade the dye partially or even totally, they have several drawbacks such as the generation of by-products, including chemical sludge, and high investment and operating costs [1].

It used to be generally accepted that carbon and nitrogen limitation favored the production of lignolytic enzymes in white rot fungi [47]. However, more recent results are somewhat contradictory. For example, in *L. edodes*, Buswell et al. [48] obtained 5-fold higher laccase levels under high nitrogen conditions than in low-nitrogen cultures, while Hatvani and Mécs [49], working on the biodegradation of dyes by *Lentinus sp.* grown in solid media, found that faster decolorization occurred at very low NH4Cl, peptone and malt extract concentrations. On the other hand, the lignolytic activity of *L. edodes* grown in liquid culture was stimulated by high N concentrations [50]. In the present work, the decolorization was affected by the type and concentration of the nitrogen source, but different trends occurred for different sources: higher concentrations of ammonium salts resulted in higher decolorization, while for sodium nitrate and urea higher production levels were obtained at the lower concentrations. Moreover, the final result varied drastically with the type of carbohydrate present in the culture medium. For example, the high decolorization obtained in cultures containing 5 g/L urea was almost lost when glucose was replaced with fructose as the main carbon source. A similar effect was observed for ammonium oxalate.

## Conclusions

*C. plicatilis* cultures were able to decolorize and biodegrade the textile dye reactive blue 19. The decolorization was highly influenced by medium composition and culture conditions, being higher in media containing intermediate concentrations of ammonium oxalate and glucose. Decolorization of dye was associated with laccase displaying good tolerance to a wide range of pH values and temperatures, suggesting a potential role for this organism and enzyme in the remediation of real dye containing effluents. In conclusion, laccase activity in *C. plicatilis* was firstly described in this study.

## Competing interests

The authors have declared no conflict of interest.

## Authors’ contribution

HAA: Designed experiments and drafted the manuscript. MCT: carried out the experiments, analyzed data. AAU: carried out the experiments. All authors have read and approved the final manuscript.
